# Anthelmintic efficacy of oxibendazole against gastrointestinal nematodes in swine

**DOI:** 10.1590/S1984-29612022009

**Published:** 2022-02-23

**Authors:** Rafael Paranhos de Mendonça, Daniela Oliveira Carneiro, Eliane Marucci Baccin, Márcia Richena Pirágine, Sara Menegatti Zoca, Luis Augusto Ferreira Rossa, Guilherme Cecílio Lima, Francismar Barbosa de Oliveira, Istanlei Soares Costa, Gabriel Nunes de Oliveira, Sabrina Nathália Louzada Nogueira, Thaís Rabelo dos Santos-Doni

**Affiliations:** 1 Universidade de Franca – UNIFRAN, Franca, SP, Brasil; 2 Médico Veterinário Autônomo, Franca, SP, Brasil; 3 Universidade Federal dos Vales do Jequitinhonha e Mucuri– UFVJM, Unaí, MG, Brasil

**Keywords:** Anthelmintic, oxibendazole, nematodes, swine, Anti-helmíntico, oxibendazol, nematódeos, suínos

## Abstract

In swine production, parasites, especially gastrointestinal helminths, generate considerable economic losses. Therefore, effective control measures, such as the use of the correct anthelmintics, are of paramount importance for maintaining profitability. The aim of the present study was to evaluate the efficacy of the anthelmintic oxibendazole, administered orally, in pigs (non-industrial) naturally infected with gastrointestinal nematodes. To that end, we selected 18 pigs naturally parasitized by gastrointestinal nematodes, as determined by examination of fecal samples (eggs per gram (EPG > 500) of feces), and divided them into two groups: treated (with a 10-day course of oxibendazole) and control (untreated). After the treatment period, the animals were euthanized. During necropsy, the helminths in the gastrointestinal tract were identified and quantified. The species identified were, in order of occurrence, *Ascaris suum*, *Trichuris suis*, *Oesophagostomum dentatum*, and *Hyostrongylus rubidus*. In Brazilian swine herds, traditional (non-industrial) production systems can favor the transmission of helminths. We found that treatment with oxibendazole was 100% effective against *A. suum* and *H. rubidus*, whereas it was 99.65% effective against *O. dentatum* and 99.20% effective against *T. suis*, significantly reducing helminth counts (P < 0.01 for all). We conclude that oxibendazole is effective in controlling the main helminths in swine.

## Resumo

A presença de parasitoses na suinocultura gera prejuízos econômicos relevantes. Dentre elas, destacam-se as helmintoses gastrintestinais. Sendo assim, medidas de controle efetivas, tais como o uso de anti-helmínticos corretos, são importantes para a lucratividade na criação de suínos. O presente estudo teve como objetivo avaliar a eficácia do oxibendazol, administrado pela via oral, em suínos (não industrial) naturalmente infectados por nematódeos gastrintestinais, na região de Franca/SP. Para isso, foram selecionados, por meio de exames coproparasitológicos (ovos por grama (OPG > 500) de fezes), 18 suínos naturalmente parasitados por nematódeos gastrintestinais, que foram divididos em dois grupos experimentais: grupo tratado (oxibendazol) e grupo controle. Após o tratamento, os animais foram submetidos à necropsia parasitológica, e os resultados das quantificações de helmintos presentes no trato gastrintestinais indicaram a presença das espécies *Ascaris suum, Trichuris suis, Oesophagostomum dentatum* e *Hyostrongylus rubidus* em ordem decrescente de ocorrência. Nos rebanhos suínos brasileiros, os sistemas de produção não tecnificadas podem favorecer a transmissão de helmintos. O tratamento com o oxibendazol obteve eficácia de 100% para as espécies *A. suum* e *H. rubidus,* de 99,65% para o *O. dentatum* e de 99,20% para o *T. suis*, reduzindo significativamente (P < 0,01) as contagens de helmintos, comprovando que o uso do oxibendazol é eficaz no controle dos principais helmintos de suínos.

Brazil is currently the fourth largest producer and exporter of pork in the world (ABCS, 2020). In recent decades, the worldwide consumption of meat, including pork, has increased markedly. In 2019, per capita pork consumption in Brazil was 15.3 kg/year (ABPA, 2020). In 2015, the biggest consumers of pork were China, the European Union, the United States, Russia, Vietnam, and Brazil, which collectively accounted for 82% of the global consumption (ABCS, 2016). However, losses in productivity are recurrent problems that are linked to the presence of parasites and other diseases in the herd, which must be rigorously studied so that producers can develop effective control measures (Lopes et al., 2014). In addition to the economic losses, the significance of helminths in swine is mainly due to the fact that many of the related zoonoses are extremely relevant and are typically neglected (Mundim et al., 2004).

Pig feces are a source of pathogenic organisms, including bacteria, viruses, parasites, and fungi (Bornay-Llinares et al., 2006). In Brazil, the most prevalent helminth species in pigs are nematodes (Lignon et al., 1998): *Ascaris suum*, *Trichuris suis*, *Hyostrongylus rubidus*, *Oesophagostomum dentatum*, *Strongyloides ransomi*, and *Metastrongylus salmi*.

Benzimidazoles have been widely used to prevent and treat parasite infections since the introduction of thiabendazole as an antimicrobial, antiprotozoal, antiviral, and anthelmintic drug (Soni, 2014). Oxibendazole, a well-known benzimidazole that contains sulfide and sulfoxide functional groups, which give it a wide range of activities, is an oral anthelmintic heterocyclic compound used in human and veterinary health care (Park et al., 2019; Papich, 2020).

One of the main methods of controlling parasitic diseases in swine production is the strategic use of anthelmintics. The efficacy of oxibendazole as an anthelmintic in pigs has been demonstrated in a study, conducted in the 1980s, in which it showed 100% efficacy in preventing intestinal worms (Fonseca & Grisi, 1989).

There have been few studies of the occurrence of intestinal parasite infection or of the use and efficacy of anthelmintic drugs in pigs in Brazil, and most such studies have not been up to date. On the basis of the above, the aim of the present study was to evaluate the anthelmintic efficacy of oral oxibendazole against gastrointestinal nematodes in naturally infected pigs, as well as to describe the occurrence of helminths in the region surrounding the city of Franca, Brazil.

This study was approved by the Animal Research Ethics Committee of the Research and Development in Veterinary Medicine Sector of the Brazilian organization Bioxen (Reference no. 010/2014). The work follows the guidelines published by the Brazilian College of Animal Experimentation and the International Guiding Principles for Biomedical Research Involving Animals established by the Council for International Organizations of Medical Sciences.

The research was carried out on the Cachoeirinha farm, which is located in the municipality of Rifaina (20°04'51”S; 47°25'15”W), in the state of São Paulo. The study was conducted in accordance with the standards of the following references: VICH GL7: Efficacy of Anthelmintics: General Requirements (Vercruysse et al., 2001); VICH GL16: Efficacy of Anthelmintics: Specific Recommendations for Porcines (Vercruysse et al., 2002); VICH GL9: Good Clinical Practice (FDA, 2001); World Association for the Advancement of Veterinary Parasitology guidelines for evaluating the efficacy of anthelmintics in swine (Hennessy et al., 2006); and the Brazilian technical regulation of antiparistic agents for veterinary use (Brasil, 1997).

For the selection process, fecal samples were collected from 220 pigs. The animals were from various non-industrial (traditional) farms in the Franca region and were identified by their ear tags. From these 220 samples, we selected 18 hybrid (*Sus scrofa domesticus*) pigs—9 males and 9 females; 2–5 months in age; and weighing 20–35 kg. All of the animals were acquired from farms with a history of intestinal worm infection and were selected by counting the eggs per gram (EPG > 500) of feces, as described by Gordon & Whitlock (1939). After the presence of helminths had been confirmed, the animals were purchased commercially and transferred to the experimental farm, where they were housed in 5.1 m^2^ stalls. Treated and control animals were housed in separate areas, with individual access to food and water, and were exposed to natural conditions of light, humidity, and temperature.

The animals were acclimatized over a period of five days, before treatment, designated day negative 5 (D−5) through day negative 1 (D−1), period which they received commercial feed *ad libitum.* Feed consumption was monitored to guarantee the ingestion of the indicated dose of the product tested. During that period, the animals were weighed to calculate the dose to be administered to each animal.

The day before the start of treatment, two animals with an EPG count > 500 were euthanized in order to identify the helminth species and stages of development, as well as to ensure the efficiency of the selection process. After those two animals had been euthanized, animal 12 died, reducing the number of animals in the sample to 15. The 15 remaining animals were divided into two groups: control (n = 7), comprising untreated pigs; and treated (n = 8), comprising pigs treated with oxibendazole.

Treatment with oral oxibendazole was carried out for ten consecutive days, designated day 0 (D0) through day 9 (D9). Stratification occurred on D0 and was determined based on sex and mean EPG count in the three days prior to treatment. The animals were listed in descending order by the mean EPG counts on D−3, D−2, and D−1 (group control - mean EPG = 633; group treated - EPG = 638). The first animal of each sex with the highest mean EPG count was randomly allocated in one of the repetitions (1 or 2), the procedure being repeated until all of the animals had been allocated, in order to compose groups that were homogeneous. Five days after the last day of treatment, each animal was euthanized and submitted to necropsy with parasitological examination.

On D0 through D9, the animals in the treated group were fed the same chow used in the acclimatization period, to which the oxibendazole was added at 20 mg of commercial product (Oxyverm® - Oxibendazol 10%, Farmabase Saúde Animal) per kilogram of body weight per day, administered orally for ten consecutive days (2 mg/kg/day). That corresponds to a daily dose of oxibendazole of 2 mg per kilogram of body weight. To ensure total consumption of the product, the animals were given only 80% of the mean quantity of feed consumed on D−3, D−2, and D−1. The diet of the animals in the control group was managed in the same way as that of those in the treated group, although it was free of any medication. The animals in both groups had *ad libitum* access to water.

The animals were humanely euthanized 15 days after the start of treatment. Procedures for organ harvesting, parasite recovery, and worm counting were in accordance with the recommendations of the World Association for the Advancement of Veterinary Parasitology (Hennessy et al., 2006), as well as with other guidelines such as those issued by the U.S. Food and Drug Administration Center for Veterinary Medicine (FDA, 2001; Vercruysse et al., 2001, 2002).

The esophagus, stomach, small intestine, and large intestine were tied with a double ligature and removed, after which the contents were carefully washed and sieved through a Tyler 48 mesh (0.297 mm) sieve, leaving only the solid contents of each segment, which were placed in appropriate containers with 10% formalin heated to 80°C (Hennessy et al., 2006). The other organs (lungs, heart, liver, pancreas, and kidneys) were also carefully examined, any and all helminths being collected (Vercruysse et al., 2002).

The counts and identification of helminth genera were performed with a stereomicroscope. The species were identified in accordance with the taxonomic criteria described previously (Costa, 2013).

The anthelmintic efficacy of the compound was calculated for arithmetic and geometric means as follows: [*control group mean* − *treated group mean*] ∕ *control group mean* × 100.

The statistical analysis was based on a comparative study between the control group and the treated group in terms of the counts for each parasite species. Prior to analysis, a logarithmic transformation [ln (x + 1)] was applied to the worm count data. The transformed values were analyzed using a general linear mixed model, including fixed (treatment group) effects and random effects. Geometric means and the corresponding 95% confidence intervals were obtained by using the transformed values. Treatment differences were assessed with the Wilcoxon rank-sum test, at a significance level of 5% (P < 0.05). Statistical calculations were performed with the Stata SE statistical software package, version 16.1 (StataCorp, College Station, TX, USA).

None of the animals employed in this study showed adverse effects during the experiment. The species found in the pigs evaluated were *A. suum*, *T. suis*, *O. dentatum*, and *H. rubidus* (Table 1).

**Table 1:** Postmortem worm counts, group means (arithmetic and geometric), and efficacy of oral oxibendazole at 2.0 mg/kg of body weight in pigs.

**Groups/**	**Gastrointestinal helminths**																							
**Identification**	***Ascaris suum***					***Trichuris suis***					***Oesophagostomum dentatum***					***Hyostrongylus rubidus***					**Total**			
	**AM**		**GM**			**AM**		**GM**			**AM**		**GM**			**AM**		**GM**			**AM**		**GM**	
**Untreated control (*n* = 7)**																								
Total parasites	284		10.63			591		10.58			523		12.23			40		5.1			1438		38.53	
Mean	40.57	^A^	31.95	^A^		84.43	^A^	31.46	^A^		74.71	^A^	54.83	^A^		5.71	^A^	4.35	^A^		205.43	^A^	319194.22	^A^
SD	27.43		0.34			109.51		0.81			62.38		0.37			3.99		0.37			107.64		1.07	
Range	10.00 - 87.00		1.04 - 1.94			0.00 – 311.00		0.00 - 2.49			16.00 – 178.00		1.23 - 2.25			0.00 - 12.00		0.00 - 1.11			70.00 – 363.00		3.41 - 6.44	
**Oxibendazole orally 2.0 mg/kg BW (*n* = 8)**																								
Total parasites	0		0			5		0.78			3		0.6			0		0			8		1.38	
Mean	0	^B^	0	^B^		0.63	^B^	0.25	^B^		0.38	^B^	0.19	^B^		0	^B^	0	^B^		1	^B^	0.49	^B^
SD	0		0			1.77		0.29			1.06		0.23			0		0			2.83		0.52	
Range	0.00 - 0.00		0.00 - 0.00			0.00 - 5.00		0.00 - 0.78			0.00 - 3.00		0.00 - 0.60			0.00 - 0.00		0.00 - 0.00			0.00 - 8.00		0.00 - 1.38	
**% Efficacy**	**100**		**100**			**99.26**		**99.2**			**99.5**		**99.65**			**100**		**100**			**99.51**		**100**	
Classification of strain	Sensitive		Sensitive			Sensitive		Sensitive			Sensitive		Sensitive			Sensitive		Sensitive			Sensitive		Sensitive	
P value	0.0004					0.0032					0.0006					0.0017					0.0006			
CV	63.47					63.47					67.20					58.67					67.20			

AM: arithmetic means; GM: geometric means; SD: standard deviation; CV: adjusted variance. Means in the same column with different superscripts (A, B) are significantly different (p < 0.01).

As a common practice, pig farmers administer anthelmintics mixed with food or water for one to ten consecutive days. The use of benzimidazoles as broad-spectrum anthelmintics in all age groups of pigs is a common practice in different regions of the world (Theodoropoulos et al., 2001; Beloeil et al., 2003).

As can be seen in Table 1, oxibendazole, administered orally for ten consecutive days, showed high (> 99.00%) efficacy against all nematodes found (P < 0.01). One of the benefits of effective anthelmintic treatment is that it reduces environmental contamination by removing female parasites, which act as sources of infection for other susceptible hosts. Anthelmintics with ovicidal activity can also minimize environmental contamination. The ovicidal activity of benzimidazole anthelmintics has been demonstrated in a wide variety of nematode species (Zhao et al., 2017).

It is important to highlight the high efficacy of oxibendazole against *O. dentatum* and *T. suis*, which are released in the intestine and migrate to the superficial mucosa of the cecum and colon, which typically makes them resistant to anthelmintic treatments (Alvarez et al., 2013; Jouvin & Kinet, 2012; Lopes et al., 2014). Similar results were obtained by Alvarez et al. (2013), who studied the anthelmintic activity of oral oxfendazole administered to naturally parasitized piglets in a single dose of 30 mg/kg of body weight, finding it to be safe and highly (100%) effective against the adult stages of *A. suum*, *Oesophagostomum* spp., *T. suis*, and *Metastrongylus* spp.

Dias et al. (2011) evaluated the effectiveness of fenbendazole 4%, at 250 g per ton of pig feed, for ten days. When *A. suum* was identified during slaughter, the farms from which the pigs came carried out a new deworming protocol, which consisted of administering the drug to all batches of animals on the farm and repeating the protocol after 20 days. The authors reported that the booster treatments were ineffective.

In Brazilian swine herds, non-industrial production systems can favor the transmission of helminths. Growing knowledge about the survival and infectivity of *A. suum* and *T. suis* eggs on pastureland indicates that they can pose a serious threat to free-range swine production. In addition, it is evident that *A. suum* is zoonotic and the same may be true for *T. suis* (Roepstorff et al., 2011). Given these new challenges and the economic impact of such infections, more research is needed. In the present study, oxibendazole was found to be highly effective against all of the helminths evaluated, providing a ≥ 99% reduction in the counts for each species and presenting a statistically significant difference (P < 0.01) when compared with the control group.

There have been few studies of the use and efficacy of anthelmintic drugs in pigs in Brazil, and most such studies have not been up to date, therefore the use of oxibendazole can be an alternative to, farmers and veterinarians that must always be aware that parasites are present, furthermore must remain permanently aware of the management system and control practices in order to limit the risk that the number of parasites will increase, which would result in production losses.

Considering the results obtained with the experimental model proposed, we can conclude that the product evaluated provides significant reductions in helminth counts among naturally parasitized swine. Oxibendazole, administered orally for ten consecutive days (2 mg/kg/day), appears to be a viable alternative for the control and treatment of pigs naturally infected with this helminths.
